# Spatial Transcriptomic Profiling of Human Saphenous Vein Exposed to Ex Vivo Arterial Haemodynamics—Implications for Coronary Artery Bypass Graft Patency and Vein Graft Disease

**DOI:** 10.3390/ijms251910368

**Published:** 2024-09-26

**Authors:** Liam W. McQueen, Shameem S. Ladak, Georgia R. Layton, Marcin Wozniak, Charles Solomon, Zein El-Dean, Gavin J. Murphy, Mustafa Zakkar

**Affiliations:** Department of Cardiovascular Sciences, University of Leicester, Leicester LE1 7RH, UK; lwm3@leicester.ac.uk (L.W.M.); ssl17@leicester.ac.uk (S.S.L.); gl186@leicester.ac.uk (G.R.L.); mw299@leicester.ac.uk (M.W.); cs806@leicester.ac.uk (C.S.); zein.eldean@uhl-tr.nhs.uk (Z.E.-D.); gjm19@leicester.ac.uk (G.J.M.)

**Keywords:** spatial transcriptomics, intimal hyperplasia, accelerated atherosclerosis, vein graft disease, haemodynamics

## Abstract

Vein graft disease is the process by which saphenous vein grafts, utilised for revascularisation during coronary artery bypass graft surgery, undergo an inflammation-driven intimal hyperplasia and accelerated atherosclerosis process in subsequent years after implantation. The role of the arterial circulation, particularly the haemodynamic properties’ impact on graft patency, have been investigated but have not to date been explored in depth at the transcriptomic level. We have undertaken the first-in-man spatial transcriptomic analysis of the long saphenous vein in response to ex vivo acute arterial haemodynamic stimulation, utilising a combination of a custom 3D-printed perfusion bioreactor and the 10X Genomics Visium Spatial Gene Expression technology. We identify a total of 413 significant genes (372 upregulated and 41 downregulated) differentially expressed in response to arterial haemodynamic conditions. These genes were associated with pathways including *NFkB*, *TNF*, *MAPK*, and *PI3K/Akt*, among others. These are established pathways involved in the initiation of an early pro-inflammatory response, leukocyte activation and adhesion signalling, tissue remodelling, and cellular differentiation. Utilising unsupervised clustering analysis, we have been able to classify subsets of the expression based on cell type and with spatial resolution. These findings allow for further characterisation of the early saphenous vein graft transcriptional landscape during the earliest stage of implantation that contributes to vein graft disease, in particular validation of pathways and druggable targets that could contribute towards the therapeutic inhibition of processes underpinning vein graft disease.

## 1. Introduction

Ischaemic heart disease (IHD) is one of the most common causes of mortality and morbidity in the world, resulting from the stenosis and occlusion of the coronary arteries supplying blood to the heart [1,2]. Management of patients with IHD includes non-invasive approaches such as modification of risk factors including hypertension and diabetes; however, revascularisation may be required in some groups of patients with more severe disease progression, via either percutaneous intervention (PCI) or coronary artery bypass graft (CABG) surgery. CABG requires the harvest and use of autologous bypass conduits to bypass diseased or occluded vessels to facilitate revascularisation, of which the most frequently used are the left internal mammary artery, the long saphenous vein (LSV), and the radial artery. The LSV is the most utilised conduit due to its ease of accessibility, handling capacity, and length, allowing for the construction of multiple conduits from the same tissue [3]. Additionally, the use of multiple arterial conduits in favour of vein grafts have not afforded consistently better outcomes over time [4].

The use of the LSV as a graft conduit is not without challenges as a considerable number of veins will gradually progress to failure over time, a process termed “vein graft disease”, in which the graft undergoes a detrimental morphological change, resulting in the stenosis and occlusion of the vessel (termed intimal hyperplasia (IH)), accompanied by an accelerated atherosclerosis process [5]. IH occurs as the result of chronic structural changes in vein graft morphology due to the abnormal migration and proliferation of smooth muscle cells (SMCs), accompanied by an increase in the amount of extracellular matrix (ECM) [6]. IH is a multifactorial process that starts during the harvesting of veins, which is known to result in the activation of endothelial cells (EC). ECs are further activated by the sudden exposure to new mechanical forces in the arterial circulation such as distension and shear stress via the activation of different signalling pathways including MAPK and NFKB [7].

To identify the impact of acute arterial haemodynamics on early vein graft gene expression and tissue remodelling, a high-resolution technique that can identify and localise transcript expression is essential to elucidate the disease process. The development of sequencing technologies such as next-generation (NGS) and single-cell sequencing have been revolutionary in facilitating a deeper understanding of the processes and interactions underpinning atherosclerosis and vein graft disease at the transcriptome level [8]. Recent research into vein graft disease pathology using these technologies have included a single-cell analysis of the role of vein progenitor cells in mice and a separate NGS sequencing analysis of progenitor cell response to cyclic strain stimulation in humans [9,10]. To date, only one single-cell analysis in humans has been undertaken that attempts to characterise cellular expression in baseline graft tissue, whilst a separate NGS study has sought to investigate transcription factor differential expression in vein grafts subjected to cyclic strain conditions [11,12]. However, no study has been undertaken that can directly quantify the transcriptomic response to acute arterial haemodynamic stimulation or cyclic strain and localise these variations to tissue/cell specific locations within the vessel [13].

Using a novel ex vivo perfusion system, in this study, we look to undertake the first-ever spatial transcriptomics analysis of the vein graft transcriptional landscape to identify the early changes in grafts in response to arterial haemodynamic conditions that can contribute to the onset of vein graft disease and localising said changes to specific regions of the tissue. We subsequently sought to investigate pathways enriched under these conditions and identify potential druggable targets.

## 2. Results

### 2.1. Whole-Tissue Differential Gene Expression in Response to Arterial Haemodynamics

**Figure 1:**
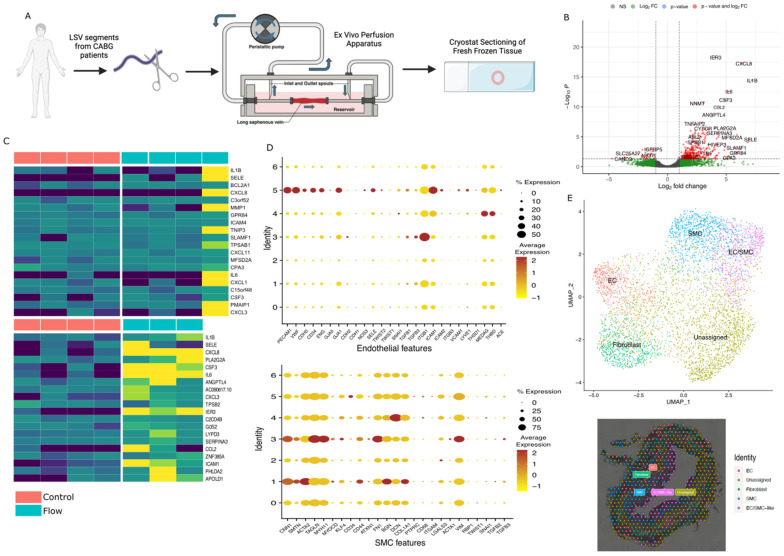
Overview of experimental process and spatial transcriptomic data analysis of whole-tissue differential gene expression. (**A**) Diagrammatic representation of the experimental protocol, including LSV harvest, ex vivo acute arterial haemodynamic stimulation, and tissue sectioning process. (**B**) Volcano plot of whole-tissue differential gene expression in response to acute arterial haemodynamic stimulation. Values from four independent experiments matched patient samples for each experimental condition. Significance values obtained from Wilcoxon rank-sum test, Benjamini–Hochberg multiple testing correction. (**C**) Heatmap of differential gene expression classified by group (control or static tissue compared against tissue exposed to acute arterial haemodynamic stimulation (dark blue indicates no/low expression, yellow indicated high expression, gradient scale). (**D**) Dot plot indicating the expression of common transcript features associated with cell types such as endothelial (EC) and smooth muscle (SMC) cells to verify cellular identity. Dot size representative of percentage of tissue spots in each cluster expressing the associated marker gene. Average expression scaled by colour (red–yellow). (**E**) UMAP plot illustrating variation between clusters, determined by shared nearest neighbour unsupervised clustering analysis and labelled by cell type based on expression profiles. Clusters are also overlaid onto a representative tissue sample to illustrate localisation of expression to histologically appropriate regions. EC/SMC-like denotes a hybrid cluster expressing both EC and SMC characteristics.

To determine the effect of acute arterial haemodynamics on the vein graft tissue transcriptome during the early stages of implantation, we exposed sections of long saphenous veins derived from four patients who underwent coronary artery bypass graft surgery. The tissue was segmented into two parts—one was kept under static conditions, whilst the other was exposed to acute arterial haemodynamic conditions for 4 h using our custom perfusion apparatus, prior to histological sectioning of the fresh-frozen tissue for probe-based spatially resolved transcriptomics using the 10X Genomics Visium technology (Figure 1A). This approach allows for differential expression analysis on an individual patient control-exposure basis, as well as allowing for integration of each patient dataset to mitigate heterogeneity within individuals, as well as providing spatial resolution of transcripts that can be used to infer cell specific expression. Across individual samples, the number of tissue-capture areas ranged from 576 to 1651, depending on the relative vessel cross section. The number of valid detected reads ranged from 84 to 200 million, and the total unique genes detected per sample ranged from 12,000 to 17,000 (Figure S1, Table S2).

Integrated bulk analysis of genes affected by arterial haemodynamics utilised a total of 10,939 detected genes, of which 383 were statistically significant (352 upregulated and 31 downregulated, adjusted *p*-value < 0.05 (Padj)). These genes are visualised as a volcano plot (Figure 1B) and as a heatmap (Figure 1C). The top 10 most significant (Padj < 0.05) upregulated and downregulated genes are summarised in Table 1, as well as based on the greatest change in log2FC expression. The annotated functions of these genes suggest their role in facilitating cellular and morphological changes in the graft tissue, facilitation of a pro-inflammatory response, and regulation of cell survival. Sensitivity analysis was performed without inclusion of flow sample 4 (Figure 1C), which had elevated mean expression levels compared to the other samples, due to a combination of variation in tissue quality/morphology and variability in RNA capture using the spatial technology. When excluded from this analysis, 342 genes are identified as significant compared to 383 with this sample included (89.2%).

The most significantly upregulated genes of note include interleukins *IL1B* and *CXCL8*, implicated as mediators of a pro-inflammatory response acting to mediate cellular proliferation, differentiation, and angiogenesis, among other functions [14,15,16,17]. This is supported by upregulation of *SELE*, *ICAM4*, and *SLAMF4* with involvement in leukocyte activation, adhesion, and general immune regulation and positive regulation of MAPK signalling, all of which have been implicated in atherosclerosis pathophysiology [18,19,20,21,22,23]. *GPR84* is a G-coupled protein receptor believed to be induced in response to inflammation, which acts as a major mediator in fibrosis pathology and facilitates urotensin activity for vasodilation, and *MMP1*, a known matrix degradation protein, is upregulated, suggesting some level of tissue remodelling is occurring under arterial conditions [24,25,26,27]. Interestingly, the genes *TNIP3* and *BCL2A1* exhibit known involvements in the NFkB signalling processes for regulation of lipopolysaccharide response and mediation of apoptosis and haemostasis, respectively [28,29,30,31].

The most downregulated genes included *CAND2* and *NR2F2-AS1*, with known functions in regulation of cell proliferation and motility as part of the PI3K/Akt signalling pathway, and competitive inhibition of TGFB, Wnt/B-catenin, and PI3K/Akt signalling pathways, respectively [32,33,34,35]. *B3GNT8* has limited cardiovascular research data but has been implicated in cancer research to regulate cell proliferation via targeting of MMPs and *TGBF1*, regulated via c-Jun [36,37]. *SLC25A27* is known to encode for mitochondrial uncoupling proteins—target effectors of NFkB c-Rel survival behaviour via mitigation of oxidative stress [38,39]. *REM1* is predominantly expressed in endothelial cell functions in cytoskeleton reorganisation and angiogenic processes [40,41]. *CARMN* generally acts on smooth muscle cells to promote their contractile phenotype, with studies the implicating deletion of this gene with exacerbated neointimal formation and smooth muscle differentiation in vascular disease [42,43].

### 2.2. Unsupervised Clustering Analysis

To further elucidate transcriptomic variation in response to acute arterial haemodynamics, dimensionality reduction and unsupervised, shared-nearest neighbour clustering analysis was undertaken to correlate gene expression to region- and cell-specific contributions. This was also utilised to account for sample-to-sample variation in gene detection and mean expression, which may skew results in favour of individual samples that had better RNA capture. This analysis identified five major distinct clusters, whose identities were inferred by a combination of cell-specific marker identification and categorisation of the most highly expressing genes within each cluster to the cell type in which they are most frequently identified (refer to Section 4). These clusters were also subsequently visualised by overlaying the clustered capture areas onto the vessel tissue image, ensuring the expression data were localised to expected regions of the tissue.

**Figure 2:**
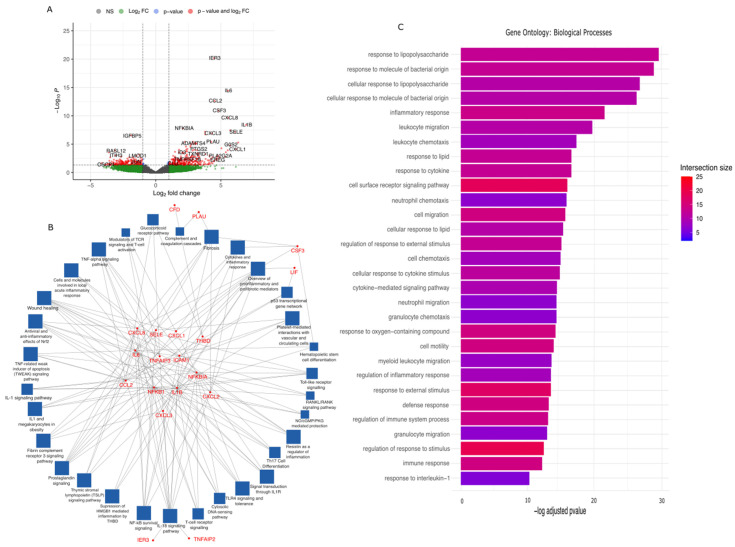
Differential gene expression and network and pathway analysis of endothelial cluster expression data. (**A**) Volcano plot illustrating the upregulated and downregulated genes activated in response to acute arterial haemodynamic stimulation. Values from four independent experiments matched patient samples for each experimental condition. Significance values obtained from Wilcoxon rank-sum test, Benjamini–Hochberg multiple testing correction. (**B**) Network analysis illustrating the pathways (and associated genes) activated within the endothelial cluster. Blue boxes represent enriched pathways, and red text represents implicated significant genes connecting these pathways. (**C**) Pathway analysis illustrating the pathways activated in the endothelial cluster. Bars are coloured based on intersection size, i.e., the number of significant genes associated with this pathway.

**Figure 3:**
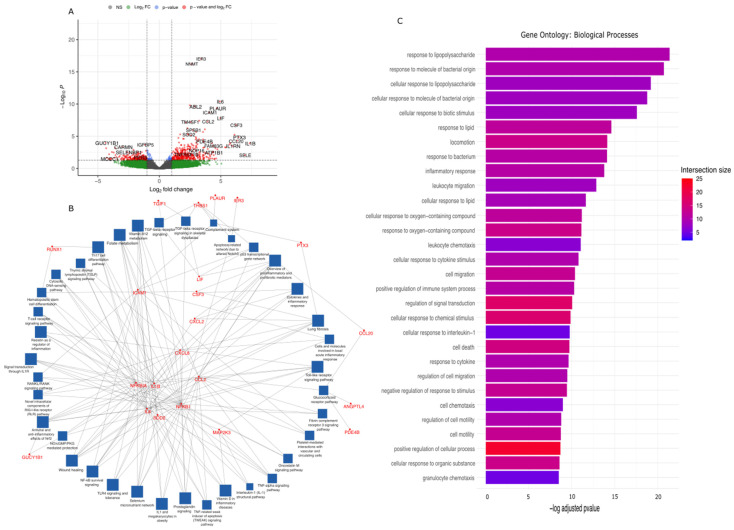
Differential gene expression; network and pathway analysis of smooth muscle cluster expression data. (**A**) Volcano plot illustrating the upregulated and downregulated genes activated in response to acute arterial haemodynamic stimulation. Values from four independent experiments matched patient samples for each experimental condition. Significance values obtained from Wilcoxon rank-sum test, Benjamini–Hochberg multiple testing correction. (**B**) Network analysis illustrating the pathways (and associated genes) activated within the smooth muscle cluster. Blue boxes represent enriched pathways; red text represents implicated significant genes connecting these pathways. (**C**) Pathway analysis illustrating the pathways activated in the smooth muscle cluster. Bars are coloured based on intersection size, i.e., the number of significant genes associated with this pathway.

**Figure 4:**
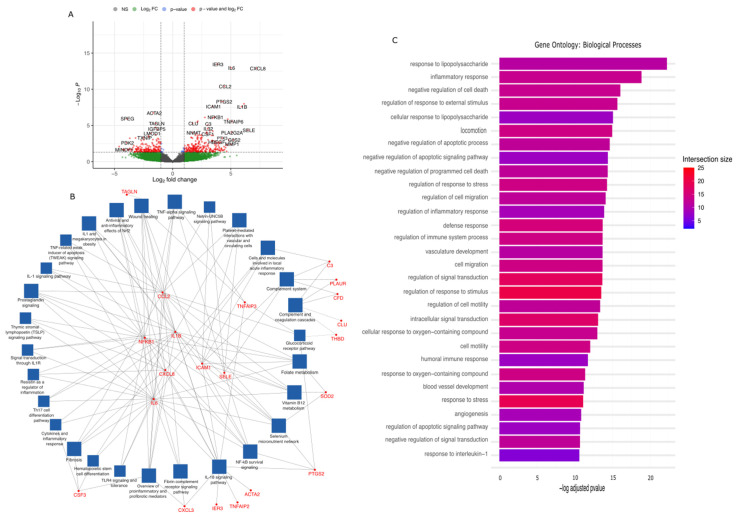
Differential gene expression, network and pathway analysis of fibroblast cluster expression data. (**A**) Volcano plot illustrating the upregulated and downregulated genes activated in response to acute arterial haemodynamic stimulation. Values from four independent experiments matched patient samples for each experimental condition. Significance values obtained from Wilcoxon rank-sum test and Benjamini–Hochberg multiple testing correction. (**B**) Network analysis illustrating the pathways (and associated genes) activated within the fibroblast cluster. Blue boxes represent enriched pathways; red text represents implicated significant genes connecting these pathways. (**C**) Pathway analysis illustrating the pathways activated in the fibroblast cluster. Bars are coloured based on intersection size, i.e., the number of significant genes associated with this pathway.

From a total of seven defined clusters, five were characterised as cell specific. These included the following: (1) endothelial cells (expression pattern 5 in Figure 1D); (2) smooth muscle cells (expression pattern 1 in Figure 1D); (3) fibroblasts (expression pattern 4 in Figure 1D); (4) endothelial and smooth muscle cells (co-expression of both cell-type markers; expression pattern 3 in Figure 1D); and (5) “unassigned” spots with mixed expression of vessel structural constituents (collagen and extracellular matrix), non-specific immune expression, mitochondrial or ribosomal genes, and genes involved in transcription and translation (expression patterns 0, 2 and 6 in Figure 1D). The endothelial, SMC, and fibroblast clusters were selected for further analysis since they are known cellular constituents of the saphenous vein. The endothelial/smooth muscle cell (EC/SMC) cluster was also selected for its similarity to cells undergoing endothelial-to-mesenchymal transition (EndMT). These clusters are visualised as uniform manifold approximation and projection (UMAP) plots (with clusters overlaid onto a representative tissue image), and key representative genes used for identification are visualised as a dot plot (Figure 1D,E), with manual interpretation of cluster expression provided in the Supplementary data (Table S1).

#### 2.2.1. Responses to Arterial Haemodynamics—Endothelial Cluster

Endothelial cells constitute the intimal layer of long saphenous veins and are responsible for facilitating numerous critical vascular regulatory aspects including responses to haemodynamic changes, homeostasis maintenance, nutrient transport, and critically, coordinating responses to inflammatory signalling [44]. From the point of harvesting to implantation during CABG surgery, the endothelial cells of the vessel are subject to surgical trauma, hypoxia, and denudation, which are further activated upon implantation in response to arterial haemodynamic conditions [45]. It is believed that these responses are the initial driver of the vein graft disease and contribute to the unresolving disease state [5].

The spots characterised as endothelial constituted 11.9% of all spots, with a total of 17,141 detected genes. Of these, 490 genes were differentially expressed in response to altered arterial haemodynamics (300 upregulated and 190 downregulated, Figure 2A), of which 29 passed false discovery rate adjustment (28 upregulated and 1 downregulated, Table 2). Genes upregulated in response to altered arterial haemodynamics included inflammatory transcripts such as *IL6*, *IL1B*, and *CXCL8* [46,47,48]. *IL6* has been suggested to act in coordination with *IL1B* and *TNF* towards VEGF-associated angiogenesis and vascular permeability in culture experiments [49]. *IER3* expression has been shown to protect cells from TNFa-induced apoptosis and has been associated with PI3K/Akt and ERK signalling [50,51]. *CSF3* is a known member of the *IL6* cytokine family, controlling the differentiation and function of monocytes/macrophages [52]. *NFKBIA* is known to inhibit NFkB/Rel complexes via cytoplasmic retention, which, upon immune and inflammatory signalling, undergoes phosphorylation, thus facilitating Rel translocation for transcription [53]. *TNFAIP3* undergoes rapid upregulation in response to TNF stimulation, acting to inhibit NFkB activation and TNF-mediated apoptosis [29]. *LIF* is involved in the promotion of cell proliferation and differentiation, interacting with its cognate receptor to activate *JAK/STAT* and MAPK signalling pathways [54]. *SELE* is explicitly expressed from endothelial cell origin, with evidence suggesting its role in the leukocyte adhesion process to endothelial surfaces in response to inflammation and is strongly implicated in atherosclerosis pathogenesis [18,19]. *IGFBP5* was the only downregulated gene that, in endothelial cells, exhibits antiangiogenic behaviour via reduction in phosphorylated Akt and eNOS levels during *VEGF*-mediated angiogenesis [55].

Pathway enrichment analysis was undertaken with significant genes (Padj < 0.05) as described in the Section 4 (Figure 2B,C, Table S3). Interactions between genes significantly upregulated in response to arterial haemodynamics and the pathways in which they are associated (as determined by WikiPathways) are visualised in Figure 2B. This includes pathways such as the TNF-alpha and TNF-related weak inducers of apoptosis (TWEAK) signalling, cytokine and interleukin signalling associated with inflammatory response, NFkB survival signalling, cell cycle regulation, and platelet-mediated interaction with vascular and circulating cells, among others. Uniquely expressed genes in this cluster include *PLAU* (associated with complement/coagulation cascades and fibrosis) and *CXCL1* (wound healing, T-cell receptor modulation, inflammatory response, and fibrosis and prostaglandin synthesis). Enriched pathways derived from gene ontology databases identified responses to lipids and lipopolysaccharide, general inflammatory response, leukocyte migration/chemotaxis, response to external stimulus, and cell population proliferation, among others (Figure 2C). These pathways are classified based on intersection size, i.e., the number of significant genes that are associated with the pathways derived from external databases, which are also present in our differentially expressed experimental data.

#### 2.2.2. Responses to Arterial Haemodynamics—Smooth Muscle Cluster

Smooth muscle cells compose the medial layer of long saphenous veins and act to maintain vessel structural integrity, but predominantly their physiological role is to respond to pressure-sensing signals from the endothelial cells of the intima to contract or relax the vessel in response to haemodynamic variation and maintain blood pressure homeostasis [56]. However, in the context of vein graft disease, the non-resolving activation of endothelial cells facilitates a phenotypic shift in smooth muscle cells, resulting in the proliferation and migration leading to intimal hyperplasia development and eventual occlusion of the graft [57].

The spots with smooth muscle gene expression patterns constituted 19.5% of all spots, with 17,372 detected transcripts. Of these, 580 genes were differentially expressed in response to the altered arterial haemodynamics (377 upregulated and 203 downregulated, Figure 3A) with 40 passing false discovery rate adjustment (39 upregulated and 1 downregulated; Table 3). Genes upregulated in response to changes in arterial haemodynamics included the previously described *IL6* and *LIF*. Others included *IER3* with a role in protection from TNF-alpha induced apoptosis, potentially via inhibition of ERK dephosphorylation and associations with the PI3K/Akt pathway [50,51]. *CCL2* (also known as *MCP1*), responsible for the induction of monocyte chemotaxis and calcium mobilisation, also increases. The cytokine has been linked to both atherosclerosis and vein graft disease, where it contributes to smooth muscle migration and proliferation in the TGFB signalling pathway [58,59]. *NNMT* is believed to be a critical regulator of epithelial-to-mesenchymal transition through Wnt/B-catenin signalling in cancer [60]. *THBS1* is known to mediate cell–cell and cell–matrix interactions, with broad functionality in inflammation, angiogenesis, cardiovascular homeostasis, and many other processes [61]. *ABL2* has been associated with processes including cell growth and survival, cytoskeletal remodelling directly in response to external stimuli, cell motility, and adhesion behaviour [62]. *PLAUR* upregulation was found to be associated with localisation and promotion of plasmin formation, tissue reorganisation, and wound healing [63]. *ICAM1* upregulation has been strongly associated with vein graft disease. It is upregulated in response to inflammatory signalling and is known to facilitate leukocyte adhesion and rolling interactions with the vessel wall. It is also believed to act as a marker of early smooth muscle phenotype switching in atherosclerosis [64]. *MEDAG* upregulation is associated with the promotion of adipocyte differentiation, lipid accumulation, and glucose uptake [65]. The only downregulated gene—*GUCY1B1*—encodes soluble guanylate cyclase, known to mediate responses to nitric oxide and oxygen by catalysing the biosynthesis of cGMP [66,67].

Pathway enrichment was undertaken with significant genes (Padj < 0.05), as before (Figure 3B,C, Table S4). Interactions between genes significantly upregulated in response to arterial haemodynamics and the pathways in which they are associated (as determined by WikiPathways) are visualised in Figure 3B. Pathways shared with the endothelial cluster include glucocorticoid receptor pathway, anti-inflammatory effects of Nrf2 and NO/cGMP/PKG mediated protection, platelet mediated interactions with vascular and circulating cells, and NFkB survival signalling, among several others. Pathways specific to this cluster includes TGFB receptor signalling (associated with *TGIF1*, *NFKB1*, *THBS1*), complement system activation (*THBS1*, *PLAUR*, *PTX3*, *ICAM1*). Genes uniquely expressed in this cluster includes *RUNX1* (associated with Th17 cell differentiation, hematopoietic stem cell differentiation, *TGIF1* (TGFB receptor signalling), *THBS1* (TGFB receptor signalling, complement system, p53 transcriptional network and apoptosis related to Notch3 alteration), *PTX3* (complement system and fibrosis), *CCL20*, *ANGPTL4* and *PDE4B* (inflammation and glucocorticoid receptor pathway), *GUCY1B1* (NO/cGMP/PKG mediated protection and anti-inflammatory effects of Nrf2), and *MAP2K3* (vitamin D in inflammatory disease and toll-like receptor signalling). Enriched pathways derived from Gene Ontology databases identified numerous shared pathways with the endothelial cluster, however uniquely identified pathways included locomotion, cellular response to oxygen containing compounds, cell migration, positive regulation of immune system processes, regulation of cell migration and cell motility, and cell death (Figure 3C). Pathways acting across both the endothelial and smooth muscle cluster data include response to lipopolysaccharide, leukocyte migration and chemotaxis, inflammatory response signalling, and response to interleukin-1.

#### 2.2.3. Responses to Arterial Haemodynamics—Fibroblast Cluster

Fibroblasts are known to compose the adventitial layer of saphenous vein grafts and have been implicated in numerous vascular disease and wound healing states to rapidly activate, proliferate, migrate, and differentiate [68]. These myofibroblasts have been shown to develop connections with extracellular matrix constituents to facilitate wound healing, primarily through synthesis of additional extracellular matrix and subsequent contraction [69]. It is believed that these myofibroblasts directly contribute to the progression of intimal hyperplasia and negative remodelling in vein graft disease, in part through their migration to the intimal/medial layers of the vessel structure, deposition of extracellular matrix which is unresolved because of chronic pro-inflammatory signalling [70].

Spots with fibroblast expression patterns composed 14.3% of all spots, with 16,561 detected transcripts. Of these, 476 transcripts were differentially expressed in response to changes in haemodynamics (275 upregulated, 201 downregulated), and twenty-seven passed FDR adjustment (22 upregulated, 5 downregulated; Table 4, Figure 4A). The significant transcripts included *CXCL8*, *IL6*, *IL1B*, *IER3*, *CCL2* and *ICAM1* and were described previously. *CXCL3* acts as a chemoattractant for neutrophils in inflammation states and may act via the PI3K/Akt signalling pathway, a mediator of cell proliferation [71]. *PTGS2* upregulation is associated with prostaglandin biosynthesis, responsible for prostanoid biosynthesis involved in inflammation and cell division [72]. *TNFAIP3* is well established as an early response gene activated in response to TNF stimulation, inhibiting NFkB activation and TNF-mediated apoptosis [29]. *NFKB1* is a transcription factor associated with the NFkB signalling pathway, functioning as signal transduction endpoint for inflammation, immunity, and other cellular processes including differentiation, cell growth and apoptosis, among others [73]. Five genes were classified as significantly downregulated in the fibroblast cluster (Padj < 0.05). These include smooth muscle actin (*ACTA2*), whose downregulation in fibroblasts may suggest transition to a more proliferative or migratory state, which may represent the myofibroblast phenotype [74]. *SPEG* (also known as *APEG-1*) has roles in development, maintenance, and function of muscle cell cytoskeletons, with its expression associated with vascular smooth muscle cell differentiation [75]. *TAGLN* expression is also similarly associated with cell differentiation, although its function is more strongly associated with actin-crosslinking for cytoskeleton organisation and interaction with calcium [76]. *IGFBP5* has been implicated in processes relating to smooth muscle proliferation and migration in atherosclerosis research [55]. *FLNA* acts similarly to *TAGLN* as an actin-binding protein encoder acting to crosslink actin filaments to membrane glycoproteins, and as such is believed to cytoskeletal remodelling to affect cell shape and migration processes [77].

Pathway enrichment analysis was undertaken for significant genes (Padj < 0.05), as before (Figure 4B,C, Table S5). Interactions between genes significantly upregulated in response to arterial haemodynamics, and the pathways in which they are associated (as determined by WikiPathways) are visualised in Figure 3B. Numerous pathways are shared across all clusters, including NFkB survival signalling, the glucocorticoid signalling pathway, prostaglandin signalling, TWEAK signalling, complement/coagulation cascades, fibrosis and wound healing, and pro-inflammatory signalling responses, among others. Pathways specific to this cluster include netrin-UNC5B signalling, which has been implicated in the regulation of Wnt and MAPK signalling to modulate cellular pluripotency, angiogenesis, cellular survival against apoptotic stimuli, and vascular endothelial cell senescence [78,79]. Genes uniquely expressed in this cluster includes *TAGLN* (associated with wound healing), *C3* (local acute inflammatory response and complement/coagulation cascades), *CLU* (complement/coagulation cascades, *PTGS2* (folate metabolism, selenium micronutrient network, and IL18 signalling), and *ACTA2* (IL18 signalling). Enriched pathways derived from gene ontology databases identified numerous shared pathways with both the endothelial and smooth muscle cell clusters; however, uniquely identified pathways included negative regulation of cell death, negative regulation of apoptotic processes and signalling pathways, regulation of response to stress, vascular development, humoral immune response, and angiogenesis. Pathways such as locomotion, cell migration, and motility are shared with the smooth muscle cluster, such as cell migration and motility, although the pathways unique to the fibroblast cluster appear to exhibit regulatory activity on these functions rather than facilitating them in the case of the smooth muscle cluster.

#### 2.2.4. Non-Specific and Pro-Inflammatory Responses to Arterial Haemodynamics

A cluster expressing a combination of endothelial and smooth muscle cell markers is localised to the inner regions of the vessel. We have investigated this specific subset in another report, including its association with endothelial-to-mesenchymal transition (EndMT), and mechanistically validated the involvement of TWIST [80]. We have also explored the cluster previously classified as “unassigned” with expression of mitochondrial/ribosomal genes, vessel structural constituent genes (including collagen and extracellular matrix synthesis), and generalised immune-, transcription-, and translation-associated genes (Table S6). Whilst we cannot completely rule out the involvement of mitochondrial and ribosomal genes in response to arterial haemodynamic conditions, as with single-cell dataset analyses, it is often inferred that detection of these transcripts is the result of cell death and is often excluded from subsequent analysis [81,82,83]. In our experimental comparison to a static control, this is particularly prevalent due to the increased shear stress applied to the vessel, which can activate apoptotic pathways. Likewise, genes encoding vessel structural proteins cannot be attributed to any cell classification by definition and are summarised efficiently as start- and endpoints of pathways such as vessel remodelling and extracellular matrix deposition from earlier analyses.

We have previously shown that there is significant activation of pro-inflammatory signalling in vein grafts in response to arterial haemodynamics, particularly via activation of NFkB and MAPK signalling at both early (45 min) and late (4 h) timepoints in other reports [84,85]. Based on these findings, we further validated our spatial transcriptomic results using different vein graft samples (n = 4) under flow conditions. In agreement with or previous reports, we note upregulation of pro-inflammatory transcripts *MCP1* and *IL8* in response to arterial stimulation. Likewise, we note the change in regulation of transcripts related to EndMT, apoptosis, angiogenesis, and coagulation that have been implicated in the vein graft IH process (*TWIST2*, *BCL2*, *VEGFA*, and *THBD*) (Figure S2). We also sought to verify that the expression of activated (phosphorylated) NFkB coupled with activation (phosphorylation) of markers p38 and pSMAD (corresponding to activation of MAPK signalling) were upregulated during the earliest stages of exposure to arterial haemodynamic stimulation (45 min) (Figure S3), not only at the later (4 h) timepoint utilised for our spatial experiment. Using immunofluorescence for detection of these protein markers, upon exposure to arterial haemodynamics, we noted increased NFkB, p38, and SMAD activation (phosphorylation) similar to what was previously reported [84,85].

#### 2.2.5. Identification of Druggable Targets

**Figure 5:**
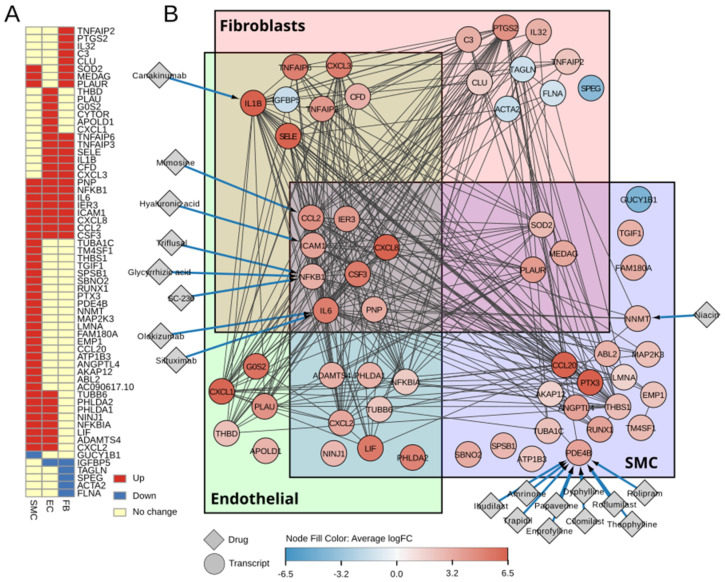
Identification of druggable targets. (**A**) Heatmap summarising expression patterns in response to shear stress in the identified clusters. Red indicates upregulation, blue represents downregulated expression, and yellow represents no change. (**B**) STRING network of all transcripts from A (circles) linked with pharmacologically active drugs (diamonds). Node colour indicates average logFC in response to shear stress in cell−type clusters. Boxes indicate expression in a specific cluster. Unboxed transcripts were significant in more than one cluster. (EC = endothelial; FB = fibroblast; SMC = smooth muscle).

Out of 62 transcripts that were differentially regulated in endothelial, smooth muscle, or fibroblast cell clusters, seven were identified as targets of pharmaceutically active drugs (Figure 5). Two targets were specific for smooth muscle cells and included *NNMT* (nicotinamide N-methyltransferase), targeted by the drug niacin, and *PDE4B* (3′,5′-cyclic-AMP phosphodiesterase 4B) targeted by 10 drugs. This included theophylline and its derivatives (dyphylline and enprofylline), selective *PDE4B* inhibitors (cilomilast and roflumilast), and other drugs indicated for cardiovascular disease (amrinone and trapidil) or patients with COPD (roflumilast and cilomilast). Other targets were differentially expressed in multiple cell clusters and mainly included molecules involved in the inflammatory response like *IL6* and *IL1B* targeted by specific monoclonal antibodies (siltuximab, olokizumab, and canakinumab) or *CCL2* (targeted by a DNA replication inhibitor, mimosine), *NFKB* (targeted by triflusal, glycyrrhizic acid and SC-236), and *ICAM1*, which binds hyaluronic acid.

## 3. Discussion

The LSV is frequently utilised as a conduit for facilitating coronary revascularisation. The use of LSV is complicated by late occlusion and restenosis that impact many utilised veins, limiting long-term benefits. Over the years and despite decades of research, we still lack a universally accepted therapeutic intervention that is capable of modulating vein graft disease and improve patency. This is related to the fact that all previous attempts focused on a single factor or pathway and did not consider the complexity of vein graft disease and the interactions between different genes and pathways. Thus, understanding the processes by which these grafts adapt (or maladapt) to the arterial haemodynamic environment is essential for improving their long-term patency and mitigating the requirement for repeated surgical intervention. In this study, we have undertaken the first in-human spatial transcriptomic analysis of LSV tissue with a view to understanding how this tissue responds to simulated arterial haemodynamic conditions like those experienced upon implantation into coronary circulation.

Analysis of the differentially expressed genes (DEGs) in our samples depicts an altered transcriptome environment for LSV graft tissue in response to acute exposure to arterial haemodynamic conditions. Within a 4-h period of simulated arterial circulation, we have identified significant pro-inflammatory activation, upregulation of processes pertaining to cellular activation, differentiation, proliferation, and migration, and activation of cell survival and wound-healing processes in response to the forces subjected to the vessel. From the whole-tissue analysis, we have also identified early indications of tissue remodelling, matrix degradation, angiogenesis, and cytoskeletal remodelling. These changes are accompanied by leukocyte activation and adhesion signalling to the tissue.

Numerous pathways identified in our analysis have been previously associated with the vein graft disease processes. The NFkB signalling pathway, for example, has been implicated in the regulation of the leukocyte adhesion cascade, involving migration of leukocytes to the endothelium and release of pro-inflammatory mediators [86]. Initiation of this pathway has been shown to result in activation of *VCAM1* and the resulting activation of Rac1 reactive oxygen species production [87]. Interestingly, *VCAM1* was found in our data to be significantly upregulated in response to arterial haemodynamics in the EC and EC/SMC clusters only (*p* < 0.05). Likewise, TGFB signalling is identified from our analysis, which has been identified as upregulated at sites of vascular injury and is implicated in the EndMT process [80,88,89]. Interactions with TGFB can result in activation of the canonical or non-canonical signalling, with the former attributed to EndMT and the latter resulting in activation of the MAPK signalling pathway resulting in the onset of vascular inflammation [5,90].

The *NR2F2* from our data was found to be downregulated in response to arterial haemodynamic stimulation in the whole tissue, whilst its associated endogenous inhibitor *NR2F2-AS1*, known for inhibition of TGFB and the Wnt/B-catenin signalling pathways, was also significantly downregulated [30,31]. Previous research has found that this marker is more significantly abundant in venous ECs, not arterial ECs, and is in direct control of maintaining the venous phenotype [91,92]. Furthermore, ECs with low expression of this gene have been shown to exhibit a more atherogenic and osteogenic phenotype, in conjunction with increased EndMT potential [92]. The PI3K/Akt signalling pathway was implicated to be upregulated in response to arterial haemodynamic conditions in our analysis—a pathway that has been implicated in the regulation of vascular tone and angiogenesis, coupled with leukocyte recruitment in ECs, and modulation of cellular proliferation [71,93]. This same pathway has been further established as a key feature of cell proliferation in vein graft neointima, acting as a regulatory pathway for SMC cell proliferation [94,95]. Given the close correlation of our transcriptomic analysis with the literature, we believe this approach to the investigation of the effects of acute arterial haemodynamic exposure is an accurate depiction of the environment in which a vein graft would be implanted.

Phosphodiesterase 4B (*PDE4B*) plays a role in the regulation of inflammatory responses, and its interaction with shear stress is particularly relevant in the context of cardiovascular and inflammatory diseases. *PDE4B* is involved in the hydrolysis of cAMP, a crucial signalling molecule that modulates inflammatory pathways. Inhibition of *PDE4B* can lead to increased levels of cAMP, which in turn reduces the production of pro-inflammatory cytokines and enhances anti-inflammatory responses. This modulation of inflammation by *PDE4B* is critical in maintaining endothelial function and preventing the progression of vascular diseases [96,97]. It is particularly relevant in areas of higher shear stress that are prone to develop inflammatory response, as suggested by our analysis. The identified drugs could offer the means to regulate the levels of cAMP, through the activity of *PDE4B*, and help control the endothelial response to biomechanical factors observed in VGD.

Of the 10 drugs identified for use in inhibiting phosphodiesterase 4 (*PDE4*) activity, these generally work by exerting their effect by mitigating hydrolysis of cAMP, increasing cAMP concentration, and reducing pro-inflammatory signalling mediators that may drive early vein graft disease onset. Several of these drugs, such as cilomilast and roflumilast, have previously only been implicated in the treatment of respiratory diseases such as asthma or chronic obstructive pulmonary disease [98,99]. Their use in cardiovascular disease beyond these conditions has not yet been explored in human trials, although evidence suggests the involvement of *PDE4* in disease states such as vascular diseases (atherosclerosis, restenosis, and cardiac hypertrophy, among others), whereby selective inhibitor of *PDE4* could be beneficial [100]. When considering the identified drugs that are actively utilised in cardiovascular disease treatment, amrinone is indicated for treatment of congestive heart failure, via inhibition of *PDE4*, *PDE3A/B*, and TNF signalling, thereby increasing cAMP/cGMP and calcium influx resulting in increased ionotropic activity [101]. Trapidil, as another example, is indicated for use in stable chronic angina, exerting both vasodilatory and anti-platelet activity. This is achieved via the broad inhibition of the phosphodiesterase protein family (including *PDE4B*), inhibition of fibroblast growth factor receptor 3 (*FGFR3*), and antagonism of platelet-derived growth factor receptor B (*PDGFRB*) [102]. It has been shown, for example, that in the context of neointimal hyperplasia and restenosis, cAMP (under the control of *PDE4*) inhibits the proliferation of SMCs, in turn limiting medial thickening [103,104]. Furthermore, studies investigating the phenotypic transition of SMCs in damaged arteries have identified a significant reliance of these phenotypically modified (synthetic) SMCs on *PDE4* activity to control cAMP, due to the drastic decreases in *PDE3* activity [105,106,107]. Inhibition of PDE4 under these circumstances, via the use of a *PDE4* inhibitor such as those we identified, may provide a means of pharmacologically mitigating against SMC proliferation and migration in bypass grafts and preventing restenosis and occlusion [108].

Spatial transcriptomics offers several unique benefits over other cutting-edge transcriptomic technologies such as single cell, particularly in the context of investigating pathologies within vascular tissue [8]. Spatial transcriptomics, as the name suggests, provides spatial resolution of expression in a manner not possible with single cell, as tissue architecture is maintained during the analysis, and cell-specific data can be referenced against the known composition of the tissue to verify the accuracy of the findings. Given that vascular disease states often result in modification of tissue morphology, and interaction with factors such as circulating pro-inflammatory signals occur at specific regions of the tissue (the endothelium), understanding the transcriptomic contributions of unique cells and regions within the tissue (as compared to the whole tissue) is key to elucidating the processes driving the onset of graft failure. This approach also allows for the determination of localised biomarker detection between cell types, and subsequent pathway analysis approaches can be utilised to, again, localise the activation of pathways to specific regions of the vasculature, thereby identifying cell and tissue areas that can be most suitably targeted by drug intervention. Previous analysis of the human long saphenous vein using a single-cell sequencing approach was undertaken by Dong et al., with the goal of identifying the cellular heterogeneity of this tissue in the context of vein graft failure [11]. Whilst this approach was uniquely useful in the identification of potentially novel EC subtypes, the lack of spatial localisation of these phenotypes, coupled with the data suggesting an EC sample composition of roughly 50% of total cells harvested from the tissue (despite the LSV comprising a single intimal monolayer), reveals a lack of the structural validation that is afforded by our approach.

The main limitation of this research approach is the lack of cell-level resolution that this technology affords—the spot-level resolution is stated to capture a region of approximately 1–50 cells according to the manufacturer, with variation dependent on tissue type, and given that the endothelial cell of the LSV is a monolayer, much of the analysis of this cell type in isolation is also likely to capture SMC transcript data. This same limitation extends to analysis of immune cell or other less frequent cells in the vessel as well given the lack of resolution and the use of only a 10 μm tissue section, much of the transcript data associated with these cell types can be indistinguishable from the rest of the transcript data or fail to reach significance due to limited expression. With that said, an improvement to this analytical approach may be to utilise a combination of spatial and single-cell transcriptomics to cross-reference cell types and infer more accurately the behaviours of individual cells, although this may be prohibitively expensive for many at this time. A further physiological limitation exists in the use of the LSV as a bypass conduit and as a model for our study—the impact of systemic, chronic inflammation on the vessel. This process can occur prior to graft excision from the patient, and the resultant pro-inflammatory signalling once implanted as a graft. We opted to utilise the LSV given its prevalent use in current operative procedures, despite the potential systemic damage that they may be exposed to.

## 4. Methods

### 4.1. Ethical Approval

Excess LSV tissue samples were obtained from anonymised, consenting patients who were undergoing coronary artery bypass graft surgery. The study was approved under the Leicester Biomedical Research Centre (BRICCS Ethics Ref: 09/H0406/114). Informed consent was obtained from all participants prior to undergoing surgery, and the use of human tissue conformed to the principles outlined in the Declaration of Helsinki [109].

### 4.2. Ex Vivo Perfusion of Saphenous Veins

Excess segments of the human long saphenous vein were taken directly from the theatre and maintained in a supplemented culture medium (RPMI 1640, 10% FBS, 100 µg/mL penicillin, and 100 U/mL streptomycin) (ThermoFisher Scientific, Bleiswijk, The Netherlands 11875093). Segments were sectioned to approximately 6 cm in length for use in ex vivo perfusion and for static/baseline controls (n = 4, respectively). Veins were cannulated with male Luer fittings 1/16” (World Precision Instruments, Sarasota, FL, USA) and secured with fine surgical tie. Attached vein segments were then placed in the perfusion apparatus, which itself was placed in a 37 °C incubator. The apparatus consisted of a closed-circuit loop system of sterile silicon tubing (VWR, Philadelphia, PA, USA, and Elkay, Rotherham, UK), with perfusion supplied using a multi-channel peristaltic pump (Watson Marlow, Falmouth UK). The tissue was perfused under mean arterial pressure (65 mmHg) with M199 media (supplemented with 20% FBS, 100 µg/mL penicillin, and 100 U/mL streptomycin), which was oxygenated and pre-warmed to 37 °C. Perfusion was designed to achieve a target wall shear stress value of 12 ± 0.2 dyn/cm^2^, which was calculated using the equation τ = 4µǪ/πr^3^, where τ represents shear stress; µ, viscosity; Ǫ, flow rate; π, pi; r, radius. These values were obtained from the diameter of the cannula, assuming shear stress through a non-deformable cylinder and assuming laminar flow and the viscosity of water. This shear stress value was chosen based on computationally derived shear stress values in the coronary artery circulation to mimic in situ conditions of the vein graft following surgical implantation. Four vein samples were utilised from patients for spatial RNA sequencing, with half of each vein section subjected to perfusion for a total of four hours, and half of the same tissue was kept under static conditions for comparison. Likewise, for validation of transcripts by comparative reverse-transcriptase polymerase chain reaction (described later), long saphenous vein graft samples from four additional patients were utilised, with perfusion undertaken for 45 min and compared to static controls as before.

### 4.3. Spatial RNA Sequencing

Spatial RNA sequencing was undertaken using the Visium Spatial Gene Expression Kit (10X Genomics, Pleasanton, CA, USA), following their established protocol. Briefly, 10 μm tissue sections were cut and mounted onto the active sequencing areas of the Visium Gene Expression slides. Tissue was fixed with ice-cold methanol, stained with haematoxylin and eosin (H&E), and imaged using an Olympus FV1000 confocal microscope (Olympus Life Sciences, Tokyo, Japan). Tissue permeabilisation was identified based on the Visium tissue optimisation protocol (10X Genomics), followed by reverse transcription (RT) using a thermocycler. This produces spatially barcoded, full-length cDNA from poly-adenylated mRNA on the slide. Second-strand synthesis was undertaken using the provided second-strand reagents, followed by denaturation and transfer of cDNA from the slide capture areas to relevant tubes for amplification. The appropriate cycle number was determined using the provided KAPA SYBR FAST qPCR master mix and cDNA primers, and the subsequent amplification utilised the Amp mix and cDNA primers. This yielded the spatially barcoded, full-length amplified cDNA required for library construction. Quality control was ensured using the Aligent Bioanalyser High-Sensitivity Kit (Aligent Technologies, Santa Clara, CA, USA). Library construction utilised a pre-determined quantity of cDNA (40 mL) with enzymatic fragmentation and size selection utilised to optimise the amplicon size. End-repair, A-tailing, adaptor ligation, and PCR were utilised to add P5, P7, i7, and i5 sample indexes, along with a TruSeq Read 2 primer sequence. RNA sequencing was outsourced and sequenced using the Illumina NovaSeq PE150 platform.

### 4.4. Spatial Transcriptomic Analysis

#### 4.4.1. Data Quality Control and Pre-Processing

Raw RNA sequencing FASTQ files were obtained following sequencing and were subjected to quality-control testing using the FASTQC (v0.12.0) and MULTIQC (v1.20) packages [110,111]. Validation of quality was followed by pre-processing using the SpaceRanger v1.3.1 pipeline provided by 10X Genomics, with sequence alignment to the 10X Genomics Human Reference Genome GRCh38-2020-A. Brightfield images in high-resolution TIFF format were manually aligned using 10X Genomics Loupe Browser 6.4.1 software, to ensure correct and accurate orientation of expression data to the tissue image. Capture areas were determined via manual selection of areas capturing tissue to minimise background detection.

#### 4.4.2. Sample Integration

Count and image data were imported and processed using R (v4.2.1) and RStudio, using the Seurat (v4.0) package [112]. Data were normalised using the SCTransform function, correcting for batch effects between sequencing runs, and expression data were filtered to exclude low-quality reads (gene counts >100 and <3500) and mitochondrial genes. All samples were integrated for joint comparisons using the Seurat SCTransform integration workflow [113], utilising common 3000 integration features and all common genes expressed between samples.

#### 4.4.3. Dimensionality Reduction and Clustering

To identify subsets within the data relating to differential expression between cell types, unsupervised graph-based clustering was undertaken using the Seurat “FindClusters” function. Data were scaled using the “ScaleData” function; principal component analysis (PCA) was performed using the “RunPCA” function with the appropriate number of principle components determined using an “ElbowPlot” function. Uniform manifold approximation and projection (UMAP) was performed for dimensional reduction using the “RunUMAP” function, shared nearest neighbours (SNN) were determined using the “FindNeighbours” function, and the “FindClusters” function was used to identify clusters based on a shared nearest neighbour modularity approach, utilising 20 principal components at a resolution of 0.8. Cluster identities were inferred by determining the relative expression of genes with established cell specificity, as well as genes associated with transition of cellular identity in the context of atherosclerotic disease [8,11,114]. This was also validated using the “FindAllMarkers” function, from which the top 30 highest expressing genes of each cluster were manually annotated with reference to the Human Cell Atlas to determine their cellular identity (Supplementary Table S1) [115]. Visualisations of these clusters were undertaken independently of the tissue using the “DimPlot” function and overlaid onto the tissue image(s) using “SpatialDimPlot”. Clustering of gene expression data can occasionally result in a larger number of detected differentially expressed genes, resulting from increased sensitivity, noise reduction, and functional enrichment within the clustered subset compared to the bulk data. However, this is coupled with possible increased false-positive discovery in terms of bias in cluster size and selection bias.

#### 4.4.4. Differential Expression Analysis

Aggregated spot expression data from all samples was tested using the muscat (v1.6) package to account for variation in spots in a multi-sample, multi-batch, multi-state experiment with group replicates [116]. Differential gene expression was determined using DESeq2 method, and contrasts between experimental groups were established using the LIMMA (v3.54.1) package and the associated “makeContrasts” function [117]. Heatmaps for significant genes were produced using the pheatmap (v1.0.12) package and volcano plots produced using the EnhancedVolcano (v1.16.0) package [118,119].

#### 4.4.5. Pathway and Network Analysis

Pathway analysis was undertaken for genes reaching significance (*p* < 0.05) using the gprofiler package and Gene Ontology: Biological Processes annotations. Network analysis was performed using the Cytoscape application (v 3.9.1) [120]. Correlation was inferred between genes of interest by running a heat diffusion analysis with standard parameters. Global and cluster-based network analysis was undertaken using default confidence and interaction parameters. Gene expression was visually quantified using a colour gradient scale, ranging from red to blue based on their log fold change expression values. Specific genes of interest were isolated from the network to identify nearest neighbouring interactors from pathways that inferred significant expression, overlap, and connections. These were used as start points for subsequent diffusion analysis using default conditions to interpret the strength of the inferred connections. The ClueGO (v2.5.9) plug-in for Cytoscape was utilised to visualise pathway-level connections in a comparable manner to the gene networks to clarify the intractability and influence of pathways on others [121].

### 4.5. Comparative Reverse-Transcriptase Polymerase Chain Reaction

Total RNA was extracted using the RNeasy mini-kit (Qiagen, Hilden, Germany, 74104). For mRNA studies, cDNA was synthesised from total RNA using the Tetro cDNA synthesis kit (Bioline, London, UK, BIO-65043). Gene expression was determined by quantitative real-time polymerase chain reaction (qRT-PCR) using a SensiFast Probe Hi-ROX kit (Bioline, BIO-82005) and gene specific primers (Table 5) on Rotor gene Q (Qiagen) using the manufacturer’s protocol. Relative levels were calculated using the 2^−(DDCt)^ method, and mRNA expression was normalised to the housekeeping gene PPIA. A list of primers utilised, with relevant data, is shown in Table 5 below.

### 4.6. Immunohistochemistry

The expression levels of specific proteins identified by transcriptomic analysis were assessed in vein sections by immunohistochemistry. Slides were incubated in pre-chilled 10% neutral buffered formalin (Sigma Aldrich, Dorset, UK, HT5014-1CS) for 15 min at 4 °C. Thereafter, slides were dehydrated in ethanol (50–100%) and allowed to dry. For staining on formalin-fixed, paraffin-embedded human samples, slides were deparaffinised and underwent antigen retrieval. Sections were then incubated in immunofluorescence blocking buffer (Cell Signalling Technology, Danvers, MA, USA, 12411) for 30 min at room temperature, followed by overnight primary antibody (Table 6) incubation in immunofluorescence antibody dilution buffer (Cell Signalling Technology, 12378) at 4 °C. Secondary antibody (HRP linked, Table 6) was applied for 1 h at room temperature followed by nuclei staining with DAPI. Control slides were routinely stained in parallel by substituting IgG or the specific IgG isotype. All images were acquired using either a Zeiss Axioscope (Carl Zeiss GmBH, Jena, Germany) with AxioVision V4.3 software or a Zeiss LSM 510 UV laser scanning confocal microscope (Carl Zeiss GmBH). The expression of proteins of interest was assessed by quantification of fluorescence intensity for multiple cells.

### 4.7. Drug Bank

Identification of druggable targets was undertaken using DrugBank (v5.1.12), a comprehensive database comprising drug and drug-target information [122]. This was utilised in conjunction with the CyTargetLinker Cytoscape plug-in (v4.1) to visualise interactions [123]. These tools can extend gene/protein networks by providing relevant associations from linked datasets, including information relating to the biochemical and pharmacological data of drugs (both clinically approved and those under investigation) that act on said gene/protein, their mechanisms of action, and their associated targets of interest.

## Figures and Tables

**Table 1:** Differentially expressed genes identified in the whole tissue by comparison of control tissue (static conditions) and flow tissue (tissue subjected to acute arterial haemodynamic perfusion). Summarisation of the top 10 upregulated and downregulated genes identified by spatial transcriptomic analysis of the whole tissue in response to acute arterial haemodynamic exposure, organised based on *p*-value significance and log fold change following multiple correction (Wilcoxon rank-sum test, Benjamini–Hochberg false discover rate correction). Values from four independent experiments matched patient samples for each experimental condition.

Top 10 Upregulated DEGs (Arranged by Significance)			
Gene	Function	Log2 Fold Change	*p*-Value (BH-Global Adjusted)
*IER3*	Functions to protect cells from TNFa induced apoptosis. May also have a role in ERK signalling via inhibiting dephosphorylation of ERK to mediate survival. Associated with PI3K/Akt signalling pathway.	4.03	4.98 × 10^19^
*CXCL8*	Commonly referred to as interleukin-8 (IL-8). Significant mediator of inflammatory response, produced by macrophages, smooth muscle, and endothelial cells (among others). Known roles in leukocyte chemotaxis and angiogenesis, and suspected roles in atherosclerosis and endothelial dysfunction.	6.4	5.26 × 10^18^
*IL1B*	Cytokine encoder produced from activated macrophages. Important mediator of inflammatory response, acting to facilitate proliferation, differentiation, and apoptosis of cells.	7.15	3.61 × 10^15^
*IL6*	Encodes for cytokine of the same name, which functions in acute inflammation response. In vessels, it acts in concert with IL1B and TNF to produce VEGF for angiogenesis and vascular permeability. Also facilitates epithelial regeneration in an inflammatory-dependent manner via IL6-YAP-NOTCH pathway.	5.19	2.55 × 10^13^
*CSF3*	Member of the IL6 family of cytokines. Controls production, differentiation, and function of leukocytes and monocytes/macrophages. Associated with ERK signalling.	4.88	6.60 × 10^12^
*APOLD1*	Encodes for endothelial cell early response proteins involved in regulation of endothelial signalling, vascular function, and (potentially) angiogenesis.	3.07	2.47 × 10^11^
*NNMT*	Methyltransferase commonly known to facilitate drug biotransformation. NNMT identified as critical regulator of epithelial-to-mesenchymal transition via suppression of Wnt/B-catenin signalling in cancer studies.	2.45	2.47 × 10^11^
*CCL2*	Also known as MCP1. Encodes for chemokine, which induces monocyte chemotaxis and intracellular calcium ion mobilisation. Implicated in monocyte recruitment in atherosclerosis and expressed by smooth muscle cells in vein graft disease to enhance their migration and proliferation. Closely associated with the TGFB signalling pathway.	4.39	1.17 × 10^10^
*ANGPTL4*	Encodes for protein responsible for regulating lipid metabolism, inhibition of proliferation, and migration and angiogenesis of endothelial cells. Known associations with IL1B production and regulation of NFkB signalling.	3.8	1.98 × 10^9^
*TGIF1*	TGFB-induced factor homeobox 1, an active transcriptional corepressor of SMAD2/3 to limit response to TGFB signalling.	2.96	3.80 × 10^8^
*IGFBP5*	Implicated in processed including response to cAMP and regulation of smooth muscle cell proliferation and migration.	−1.32	0.00116
*SPEG*	Also known as APEG-1. Involved in development of muscle cell cytoskeleton, maintenance, and function. Expression indicative of vascular smooth muscle cell differentiation.	−1.78	0.00124
*YPEL3*	Precited to enable metal ion binding; involvement in regulation of cellular senescence and in proliferation and apoptosis of myeloid precursor cells. Suspected downstream activation in response to p53 activation.	−2.14	0.00149
*NR2F2*	Encodes for member of steroid thyroid hormone family of nuclear receptors. Regulates processes including organogenesis, embryonic cellular differentiation, and metabolic homeostasis. Upregulation of NR2F2 associated with suppression of TGFB-induced epithelial-to-mesenchymal transition.	−1.3	0.00265
*GUCY1A1*	Encodes for soluble guanylate cyclase, which catalyses conversion of GTP to cGMP. Functions as receptor for nitric oxide for vasodilation.	−1.87	0.00471
*ZBTB16*	Action as a transcriptional repressor, mediated via recruitment of histone deacetylases. May play a role in development, maturation, and maintenance of myeloid and other differentiated tissues.	−1.39	0.00613
*CARMN*	Smooth muscle cell specific long non-coding RNA, which promotes their contractile phenotype. Deletion of CARMN exacerbates neointima formation and smooth muscle cell de-differentiation in vascular disease models.	−2.5	0.00716
*PDE5A*	Encodes for cGMP-binding phosphodiesterase, which hydrolyses cGMP to 5-GMP. Involvement in smooth muscle relaxation in regions of the cardiovascular system.	−1.66	0.00743
*SLC25A27*	Encodes for mitochondrial uncoupling proteins (MUPs), which function to separate oxidative phosphorylation from ATP synthesis. MUPs identified as target effectors of the NFkB c-Rel pro-survival pathway by mitigation of oxidative stress.	−3.28	0.0103
*TGFB1|1*	Functions as molecular adaptor coordinating protein–protein interactions at focal adhesion complex. Links intracellular signalling modules to plasma membrane receptors; regulates Wnt and TGFB signalling pathways.	−1.43	0.0112
*IL1B*	Cytokine encoder produced from activated macrophages. Important mediator of inflammatory response, acting to facilitate proliferation, differentiation, and apoptosis of cells.	7.15	3.61 × 10^15^
*SELE*	Transcribes for a protein deriving from stimulated endothelial cells; believed to be responsible for adhesion of circulating leukocytes to endothelial surfaces in response to inflammation. Associated with atherosclerosis pathogenesis.	7	2.61 × 10^5^
*BCL2A1*	Transcribes for protein responsible for apoptosis regulation, with functions ranging from embryogenesis to homeostasis. Direct transcription target of NFkB in response to inflammatory mediators.	6.82	8.38 × 10^5^
*CXCL8*	Commonly referred to as interleukin-8 (IL-8). Significant mediator of inflammatory response; produced by macrophages, smooth muscle, and endothelial cells (among others). Known roles in leukocyte chemotaxis and angiogenesis, and suspected roles in atherosclerosis and endothelial dysfunction.	6.4	5.26 × 10^18^
*C3orf52*	Limited data related to cardiovascular function or disease. Believed to be involved in the production of lysophosphatidic acid, which has a wide variety of functions ranging from cell proliferation, migration, gap-junction behaviour, and pro-angiogenic factor production.	6.19	0.00114
*MMP1*	Encodes for matrix metalloproteinase members. Breakdown of extracellular matrix for tissue remodelling, immune response, and wound healing.	5.9	4.87 × 10^6^
*GPR84*	G-coupled protein receptor encoder, hypothesised to enable urotensin II receptor activity (potent vasodilation). Believed to be induced in response to inflammation and as a major mediator of pathological fibrotic pathways.	5.9	0.00464
*ICAM4*	Paralog of ICAM1. Known ligand for leukocyte adhesion protein LFA1 and monocyte/macrophage-specific Cd11c/CD18.	5.86	0.00519
*TNIP3*	Involvement in cellular response to lipopolysaccharide and negative regulation of NFkB signalling.	5.82	0.00806
*SLAMF1*	Numerous functions relating to modulation of immune cell activation and differentiation. These include negative regulation of CD40 signalling and positive regulation of MAPK signalling cascade.	5.62	0.000643
*B3GNT8*	Enables N-acetylglucosaminyltransferase activity. Associated with roles in regulation of cell proliferation in cancer via targeting of MMPs and TGFB1 and regulated by c-Jun transcription factor.	−4.73	0.0438
*CAND2*	Predicted role in TATA-binding protein activity and SCF-complex assembly. Positively associated with mTOR activity in cardiac hypertrophy studies, with mTOR known to regulate cell proliferation, motility, and survival as part of the PI3K-Akt signalling pathway.	−3.88	0.0472
*LINC02388*	No established functions. Identified primarily via genome-wide association studies.	−3.67	0.0265
*NR2F2-AS1*	Acts as a competitive endogenous RNA for several microRNAs in numerous signalling pathways including TGFB, Wnt/B-catenin, and PI3K/Akt. Shares common promoter region with NR2F2.	−3.65	0.0324
*SLC25A27*	Encodes for mitochondrial uncoupling proteins (MUPs), which function to separate oxidative phosphorylation from ATP synthesis. MUPs identified as target effectors of the NFkB c-Rel pro-survival pathway by mitigation of oxidative stress.	−3.28	0.0103
*REM1*	Encodes for a GTPase member of an RAS-like GTP-binding protein family. Expressed primarily in endothelial cells for actin cytoskeletal reorganisation and endothelial cell sprouting (angiogenesis).	−3.04	0.0472
*LINC01409*	No established functions. Identified primarily via genome-wide association studies.	−2.98	0.0546
*ATG10*	Encodes for an E2-like enzyme involved in 2 ubiquitin-like modifications essential for autophagosome formation. Shown to be negatively regulated by SNHG1 and Bhlhe40, which have been associated with aortic smooth muscle cell calcification/senescence and macrophage self-renewal.	−2.82	0.0522
*CARMN*	Smooth-muscle-cell-specific long non-coding RNA, which promotes their contractile phenotype. Deletion of CARMN exacerbates neointima formation and smooth muscle cell de-differentiation in vascular disease models.	−2.5	0.00716
*KLHDC8B*	Limited data related to cardiovascular function or disease. Believed to be an essential component in maintaining mitotic integrity and maintenance of chromosomal activity.	−2.48	0.0539

**Table 2:** Differentially expressed genes identified in the endothelial cluster subset by comparison of control tissue (static conditions) and flow tissue (tissue subjected to acute arterial haemodynamic perfusion). Summarisation of the top 10 upregulated and downregulated genes identified by spatial transcriptomic analysis of the endothelial cluster in response to acute arterial haemodynamic exposure, organised based on *p*-value significance and log fold change following multiple correction (Wilcoxon rank-sum test, Benjamini–Hochberg false discover rate correction). Values from four independent experiments matched patient samples for each experimental condition.

Top 10 Upregulated DEGs				
Gene	Function	Log2 Fold Change	*p*-Value (Unadjusted)	*p*-Value (BH Adjusted)
*IER3*	Functions to protect cells from TNFa-induced apoptosis. May also have a role in ERK signalling via inhibiting dephosphorylation of ERK to mediate survival. Associated with PI3K/Akt signalling pathway.	4.48	6.72 × 10^21^	1.15 × 10^16^
*IL6*	Encodes for cytokine of the same name, which functions in acute inflammation response. In vessels, it acts in concert with IL1B and TNF to produce VEGF for angiogenesis and vascular permeability. Also facilitates epithelial regeneration in an inflammatory-dependent manner via IL6-YAP-NOTCH pathway.	5.55	3.63 × 10^15^	3.11 × 10^11^
*CCL2*	Also known as MCP1. Encodes for chemokine, which induces monocyte chemotaxis and intracellular calcium ion mobilisation. Implicated in monocyte recruitment in atherosclerosis and expressed by smooth muscle cells in vein graft disease to enhance their migration and proliferation. Closely associated with the TGFB signalling pathway.	4.52	2.26 × 10^13^	1.29 × 10^9^
*CSF3*	Member of the IL6 family of cytokines. Controls production, differentiation, and function of leukocytes and monocytes/macrophages. Associated with ERK signalling.	4.82	1.22 × 10^11^	5.25 × 10^8^
*CXCL8*	Commonly referred to as interleukin-8 (IL-8). Significant mediator of inflammatory response, produced by macrophages, smooth muscle, and endothelial cells (among others). Known roles in leukocyte chemotaxis and angiogenesis, and suspected roles in atherosclerosis and endothelial dysfunction.	5.59	2.29 × 10^10^	7.84 × 10^7^
*IL1B*	Cytokine encoder produced from activated macrophages. Important mediator of inflammatory response, acting to facilitate proliferation, differentiation, and apoptosis of cells.	6.93	4.04 × 10^9^	1.15 × 10^5^
*NFKBIA*	Inhibits the activity of NF-kappa-B/REL complexes by trapping REL dimers in the cytoplasm. Upon immune and pro-inflammatory responses, becomes phosphorylated, promoting ubiquitination and degradation, enabling REL to translocate to the nucleus and activate transcription.	2.09	1.60 × 10^8^	3.91 × 10^5^
*LIF*	Involved in growth promotion and cell differentiation of numerous target cells. Shown to interact with specific LIF receptor, leading to activation of JAK/STAT and MAPK signalling pathways.	5.72	3.59 × 10^8^	7.69 × 10^5^
*TNFAIP3*	Gene rapidly expressed in response to TNF stimulation. Acts to inhibit NFkB activation and TNF-mediated apoptosis. Has ubiquitin ligase and deubiquitinase activities and is generally involved in cytokine-mediated immune and inflammatory responses.	3.78	6.67 × 10^8^	0.000125
*SELE*	Transcribes for a protein deriving from stimulated endothelial cells; believed to be responsible for adhesion of circulating leukocytes to endothelial surfaces in response to inflammation. Associated with atherosclerosis pathogenesis.	6.1	7.28 × 10^8^	0.000125
*IGFBP5*	Implicated in processes including response to cAMP and regulation of smooth muscle cell proliferation and migration.	−1.89	2.94 × 10^7^	0.000387
*RASL12*	Ras-like family 12 protein with predicted involvement in GDP- and GTP-binding activity and GTPase activity. Predicted to be involved in signal transduction.	−3.14	0.000131	0.0644
*YPEL3*	Precited to enable metal ion binding; involvement in regulation of cellular senescence and in proliferation and apoptosis of myeloid precursor cells. Suspected downstream activation in response to p53 activation.	−3.6	0.000288	0.117
*ACTA2*	Encodes for smooth muscle actin protein; involved in cell motility, structure, integrity, and intercellular signalling. Involvement in vascular contractility and blood pressure homeostasis.	−1.27	0.000689	0.236
*ITIH3*	Encodes for heavy-chain subunit of the pre-alpha-trypsin inhibitor complex. This complex may stabilise the extracellular matrix through its ability to bind hyaluronic acid.	−3.11	0.000991	0.317
*LMOD1*	Expressed in numerous tissues; required for proper contractility of smooth muscle cells and mediates nucleation of actin filaments.	−1.47	0.00118	0.347
*SPEG*	Also known as APEG-1. Involved in development of muscle cell cytoskeleton, maintenance, and function. Expression indicative of vascular smooth muscle cell differentiation.	−2.65	0.00162	0.418
*TGFB1I1*	Functions as molecular adaptor coordinating protein-protein interactions at focal adhesion complex. Links intracellular signalling modules to plasma membrane receptors; regulates Wnt and TGFB signalling pathways.	−2.04	0.00214	0.48
*MCU*	Mitochondrial inner membrane calcium uniporter that mediates calcium uptake into mitochondria. Acts as a key regulator of short-term mitochondrial calcium loading underlying a ‘fight-or-flight’ response during acute stress, among other calcium-dependent roles.	−3.49	0.00215	0.48
*ZBTB16*	Action as a transcriptional repressor, mediated via recruitment of histone deacetylases. May play a role in development, maturation, and maintenance of myeloid and other differentiated tissues.	−1.78	0.00225	0.494

**Table 3:** Differentially expressed genes identified in the smooth muscle cluster subset by comparison of control tissue (static conditions) and flow tissue (tissue subjected to acute arterial haemodynamic perfusion). Summarisation of the top 10 upregulated and downregulated genes identified by spatial transcriptomic analysis of the smooth muscle cluster in response to acute arterial haemodynamic exposure, organised based on *p*-value significance and log fold change following multiple correction (Wilcoxon rank-sum test, Benjamini–Hochberg false discover rate correction). Values from four independent experiments matched patient samples for each experimental condition.

Top 10 Upregulated DEGs				
Gene	Function	Log2 Fold Change	*p*-Value (Unadjusted)	*p*-Value (BH Adjusted)
*IER3*	Functions to protect cells from TNFa induced apoptosis. May also have a role in ERK signalling via inhibiting dephosphorylation of ERK to mediate survival. Associated with PI3K/Akt signalling pathway.	3.4	9.83 × 10^18^	1.71 × 10^13^
*NNMT*	Methyltransferase commonly known to facilitate drug biotransformation. NNMT identified as critical regulator of epithelial-to-mesenchymal transition via suppression of Wnt/B-catenin signalling in cancer studies.	2.64	6.15 × 10^1^7	5.34 × 10^13^
*IL6*	Encodes for cytokine of the same name which functions in acute inflammation response. In vessels it acts in concert with IL1B and TNF to produce VEGF for angiogenesis and vascular permeability. Also facilitates epithelial regeneration in an inflammatory dependent manner via IL6-YAP-NOTCH pathway.	4.92	3.96 × 10^11^	2.30 × 10^7^
*THBS1*	Adhesive glycoprotein that mediates cell-to-cell and cell-to-matrix interactions. Multifunctional, with involvement in inflammation, angiogenesis, wound healing, reactive oxygen species signalling, nitrous oxide signalling, apoptosis, and cardiovascular homeostasis.	2.48	1.59 × 10^10^	6.91 × 10^7^
*ABL2*	Non-receptor tyrosine-protein kinase involved in processes linked to cell growth and survival such as cytoskeleton remodelling in response to extracellular stimuli, cell motility and adhesion, and receptor endocytosis.	2.88	2.43 × 10^10^	8.45 × 10^7^
*PLAUR*	Acts as a receptor for urokinase plasminogen activator. Plays a role in localising and promoting plasmin formation.	4.66	4.68 × 10^10^	1.36 × 10^6^
*ICAM1*	Cell surface glycoprotein expressed at a low level in immune and endothelial cells, which is upregulated in response to inflammatory stimulation. Regulates leukocyte rolling and adhesive interactions with the vessel wall. Identified as marker of smooth muscle phenotype switching in atherosclerotic plaques.	4.08	2.06 × 10^9^	5.12 × 10^6^
*LIF*	Involved in growth promotion and cell differentiation of numerous target cells. Shown to interact with specific LIF receptor, leading to activation of JAK/STAT and MAPK signalling pathways.	4.96	1.50 × 10^8^	3.25 × 10^5^
*MEDAG*	Involved in processes that promote adipocyte differentiation, lipid accumulation, and glucose uptake.	3.25	3.84 × 10^8^	0.000074
*CCL2*	Also known as MCP1. Encodes for chemokine which induces monocyte chemotaxis and intracellular calcium ion mobilisation. Implicated in monocyte recruitment in atherosclerosis and expressed by smooth muscle cells in vein graft disease to enhance their migration and proliferation. Closely associated with the TGFB signalling pathway.	3.94	5.25 × 10^8^	0.0000913
*GUCY1B1*	Mediates responses to nitric oxide (NO) by catalysing the biosynthesis of the signalling molecule cGMP	−4.38	1.06 × 10^4^	0.0473
*IGFBP5*	Implicated in processed including response to cAMP and regulation of smooth muscle cell proliferation and migration.	−1.18	0.000207	0.0767
*CARMN*	Smooth muscle cell specific long non-coding RNA which promotes their contractile phenotype. Deletion of CARMN exacerbates neointima formation and smooth muscle cell de-differentiation in vascular disease models.	−3.02	0.000462	0.129
*CAMKK1*	Calcium/calmodulin-dependent protein kinase proposed to belong to a calcium-triggered signalling cascade involved in several cellular processes. Involved in regulating cell apoptosis.	−3.59	0.000691	0.165
*MIR99AHG*	Limited functional data relating to this gene. From cancer cell line studies, may MIR99AHG act as a competing exogenous RNA for miR-577, activating the FOXP1 mediated Wnt/B-catenin pathway.	−4.33	0.000729	0.167
*LZTS2*	Function in transcription regulation and cell cycle control. Can repress beta-catenin mediated transcriptional activation and is a negative regulator of the Wnt signalling pathway.	−2.11	0.00129	0.236
*LMOD1*	Expressed in numerous tissues, required for proper contractility of smooth muscle cells and mediates nucleation of actin filaments.	−1	0.00143	0.251
*RBPMS*	Limited data in human cardiovascular disease models. In mice, predicted to be involved in positive regulation of SMAD protein signal transduction; positive regulation of pathway-restricted SMAD protein phosphorylation; and response to oxidative stress.	−1.09	0.00182	0.297
*VCL*	Codes for F-actin-binding protein involved in cell-matrix adhesion and cell-cell adhesion. Regulates cell surface E-cadherin expression and potentiates mechanosensing by the E-cadherin complex. May also play important roles in cell morphology and locomotion.	−0.937	0.00183	0.297
*GSTA4*	Encodes for form of glutathione S-transferase, involved in cellular defence against toxic, carcinogenic, and pharmacologically active electrophilic compounds. Specifically enzyme class has glutathione peroxidase activity that function in the detoxification of lipid peroxidation products.	−3.86	0.00281	0.407

**Table 4:** Differentially expressed genes identified in the fibroblast cluster subset by comparison of control tissue (static conditions) and flow tissue (tissue subjected to acute arterial haemodynamic perfusion). Summarisation of the top 10 upregulated and downregulated genes identified by spatial transcriptomic analysis of the fibroblast cluster in response to acute arterial haemodynamic exposure, organised based on *p*-value significance and log fold change following multiple correction (Wilcoxon rank-sum test and Benjamini–Hochberg false discover rate correction). Values from four independent experiments matched patient samples for each experimental condition.

Top 10 Upregulated DEGs			
Gene	Function	Log2 Fold Change	*p*-Value (BH-Global Adjusted)
*IER3*	Functions to protect cells from TNFa induced apoptosis. May also have a role in ERK signalling via inhibiting dephosphorylation of ERK to mediate survival. Associated with PI3K/Akt signalling pathway.	3.83	5.72 × 10^10^
*CXCL8*	Commonly referred to as interleukin-8 (IL-8). Significant mediator of inflammatory response; produced by macrophages, smooth muscle, and endothelial cells (among others). Known roles in leukocyte chemotaxis and angiogenesis, and suspected roles in atherosclerosis and endothelial dysfunction.	7.23	6.99 × 10^10^
*IL6*	Encodes for cytokine of the same name, which functions in acute inflammation response. In vessels, it acts in concert with IL1B and TNF to produce VEGF for angiogenesis and vascular permeability. Also facilitates epithelial regeneration in an inflammatory-dependent manner via IL6-YAP-NOTCH pathway.	5.03	6.99 × 10^10^
*CCL2*	Also known as MCP1. Encodes for chemokine, which induces monocyte chemotaxis and intracellular calcium ion mobilisation. Implicated in monocyte recruitment in atherosclerosis and expressed by smooth muscle cells in vein graft disease to enhance their migration and proliferation. Closely associated with the TGFB signalling pathway.	4.44	1.63 × 10^7^
*PTGS2*	Encodes for main enzyme in prostaglandin biosynthesis, acting as a dioxygenase and peroxidase. Responsible for prostanoid biosynthesis involved in inflammation and mitogenesis.	4.34	1.44 × 10^5^
*TNFAIP3*	Gene rapidly expressed in response to TNF stimulation. Acts to inhibit NFkB activation and TNF-mediated apoptosis. Has ubiquitin ligase and deubiquitinase activities and is generally involved in cytokine-mediated immune and inflammatory responses.	4.17	1.44 × 10^5^
*CXCL3*	Encodes for a secreted growth factor that signals through the G-protein coupled receptor, CXCR2. This protein plays a role in inflammation and as a chemoattractant for neutrophils.	6.13	2.43 × 10^5^
*IL1B*	Cytokine encoder produced from activated macrophages. Important mediator of inflammatory response, acting to facilitate proliferation, differentiation, and apoptosis of cells.	5.91	4.69 × 10^5^
*ICAM1*	Cell surface glycoprotein expressed at a low level in immune and endothelial cells, which is upregulated in response to inflammatory stimulation. Regulates leukocyte rolling and adhesive interactions with the vessel wall. Identified as marker of smooth muscle phenotype switching in atherosclerotic plaques.	3.43	4.69 × 10^5^
*NFKB1*	Pleiotropic transcription factor presents in almost all cell types and is the endpoint of a series of signal transduction events that are initiated by biological processes such as inflammation, immunity, differentiation, cell growth, and apoptosis, among others.	3.59	1.05 × 10^3^
*ACTA2*	Encodes for smooth muscle actin protein involved in cell motility, structure, integrity, and intercellular signalling. Involvement in vascular contractility and blood pressure homeostasis.	−1.67	0.000307
*SPEG*	Also known as APEG-1. Involved in development of muscle cell cytoskeleton, maintenance, and function. Expression indicative of vascular smooth muscle cell differentiation.	−3.91	0.00151
*TAGLN*	Encodes an actin-binding protein belonging to the calponin family. It is ubiquitously expressed in vascular and visceral smooth muscle and is an early marker of smooth muscle differentiation. Involved in calcium interactions and contractile properties of cells that may contribute to replicative senescence.	−1.46	0.00425
*IGFBP5*	Implicated in processes including response to cAMP and regulation of smooth muscle cell proliferation and migration.	−1.46	0.0143
*FLNA*	Protein encoded by this gene is an actin-binding protein that crosslinks actin filaments and links actin filaments to membrane glycoproteins. Involved in remodelling the cytoskeleton to effect changes in cell shape and migration. Plays a role in cell–cell contacts and adherens junctions during the development of blood vessels.	−1.3	0.0253
*LMOD1*	Expressed in numerous tissues; required for proper contractility of smooth muscle cells and mediates nucleation of actin filaments.	−1.86	0.0616
*TPM1*	Encodes for actin-binding proteins involved in the contractile system of striated and smooth muscles and the cytoskeleton of non-muscle cells.	−1.47	0.09
*EHBP1L1*	Predicted to be involved in actin cytoskeleton organisation.	−2.08	0.111
*MUSTN1*	Predicted to be involved in several processes; expressed in the major tissues of the musculoskeletal system: bone, cartilage, skeletal muscle, and tendon. Expression has been associated with embryonic development and regeneration of bone and skeletal muscle, as well as chondrocyte differentiation and proliferation.	−2.05	0.149
*TXNIP*	Major regulator of cellular redox signalling, which protects cells from oxidative stress. Inhibits antioxidative function of thioredoxin resulting in the accumulation of reactive oxygen species and cellular stress. Also functions as a regulator of cellular metabolism and of endoplasmic reticulum (ER) stress.	−2.5	0.164

**Table 5:** List of primers utilised for validation of transcriptomic data.

Primers	Manufacturer	Assay ID
PPIA	ThermoFisher Scientific	Hs99999904_m1
TWIST2	ThermoFisher Scientific	Hs00382379_m1
MCP-1	ThermoFisher Scientific	Hs00234140_m1
IL-8	ThermoFisher Scientific	Hs00174103_m1
BCL2	ThermoFisher Scientific	Hs00608023_m1
VEGFA	ThermoFisher Scientific	Hs00900055_m1
THBD	ThermoFisher Scientific	Hs00264920_s1
NR3C1	ThermoFisher Scientific	Hs00353740_m1

**Table 6:** List of antibodies utilised for validation of transcriptomic data.

Antibodies	Manufacturer	Product Code	Working Dilution
Anti-Phospho-p38 MAPK (Thr180/Tyr182) (D3F9) XP^®^	Cell Signalling Technology	4511	1:100
Anti- NFκB p65 (F-6)	Santa Cruz Biotechnology (Dallas, TX, USA)	sc-8008	1:100
Anti-Phospho-SMAD2/3	R&D Systems (Minneapolis, MN, USA)	MAB8935	1:100
Anti-VE-cadherin Rabbit Polyclonal Antibody	Invitrogen (Waltham, MA, USA)	10586453	1:100
Anti-VE-cadherin Mouse Monoclonal Antibody (16B1)	Invitrogen	14-1449-82	1:100
Anti-CD31 Rabbit Polyclonal Antibody	Invitrogen	PA5-16301	1:50
Anti-CD31 Monoclonal Mouse Antibody	R&D Systems	BBA7	1:50
Anti-Mouse IgG (H+L) Antibody, Alexa Fluor™ 568	Invitrogen	A-11031	1:100
Anti-Rabbit IgG (H+L) Antibody, Alexa Fluor™ 568	Invitrogen	A-11011	1:100
Anti-Rabbit IgG (H+L) Antibody, Alexa Fluor™ 488	Invitrogen	A-11008	1:100
Anti-Mouse IgG (H+L) Antibody, Alexa Fluor™ 488	Invitrogen	A-11001	1:100
Anti-goat IgG, HRP-linked	Santa Cruz Biotechnology	sc-2354	1:100
Anti-rabbit IgG, HRP-linked	Cell Signalling Technology	7074	1:100

## Data Availability

The raw data supporting the conclusions of this article will be made available by the authors on request.

## Supplementary Materials

The following supporting information can be downloaded at https://www.mdpi.com/article/10.3390/ijms251910368/s1.
