# Supplementary material for: Spatial Transcriptomic Profiling of Human Saphenous Vein Exposed to Ex Vivo Arterial Haemodynamics—Implications for Coronary Artery Bypass Graft Patency and Vein Graft Disease
Source: Int J Mol Sci. 2024 Sep 26;25(19):10368. doi: 10.3390/ijms251910368 (PMC11476946; doi:10.3390/ijms251910368)
Supplement: Supplementary file 1 [file ijms-25-10368-s001.zip › Supplementary Figure and Table Legends.pdf]

Supplementary Figure Legends

Supplementary Figure S1: Visualisation of spatial transcriptomic quality metrics. (A) Bar chart visualisation of genes and unique molecular identifiers (UMIs) per array spot of the Visium 10X Genomics spatial slide (n=4 control and flow samples, matched patient tissue). (B) Correlation of normalised expression data compared with the number of detected genes from raw count data per sample (n_Count_Spatial).

Supplementary Figure S2: Quantification of reverse transcriptase polymerase chain reaction (RT-PCR) fold change expression of selected markers from spatial transcriptomic data. Values from four independent experiments and mean values are shown. (TWIST2 = Twist Family BHLH Transcription Factor 2; MCP-1 = Monocyte Chemotactic Protein-1; IL-8 = Interleukin-8; BCL2 = BCL2 Apoptosis Regulator; VEGFA = Vascular Endothelial Growth Factor Alpha; THBD = Thrombomodulin; NR3C1 = Nuclear Receptor Subfamily 3 Group C Member 1). ($p < 0.05$ = *; $p < 0.01$ = **; $p < 0.001$, paired t-test).

Supplementary Figure S3: Immunofluorescence imaging of pNFkB, pp38 and pSMAD (red) expression in long saphenous vein tissue cross section, co-stained with endothelial marker CD31 (green) for determination of expression of these marker proteins in response to short (45 minute) exposure to acute arterial haemodynamic conditions using specific antibodies. Representative tissue images, values from 4 independent experiments, and mean values of quantified fluorescent intensity shown. Image scale 20□m. (NFkB = Nuclear Factor Kappa Beta; pNFkB = phosphorylated NFkB; pp38 = phosphorylated p38; pSMAD = phosphorylated SMAD; CD31 = Platelet and Endothelial Cell Adhesion Molecule 1 (PECAM1). ($p < 0.05$ = *; $p < 0.01$ = **; $p < 0.001$, paired t-test).

Supplementary Table Legends

Supplementary Table S1: Clustering annotation table (and associated figures) derived from the 'FindAllMarkers' function. Genes are provided for each cluster, alongside their associated significance values, average log fold change, tissue positions and associated cluster number. Summary tables provide classification of the top 20 most significant genes within each cluster to aid in identification of their most likely cell classifications.

Supplementary Table S2: Sequencing quality metrics derived from the 10X Genomics SpaceRanger pipeline. Samples are classified separate to quantify the quality of sequencing, as well as summarisation of factors such as the number of spots under tissue, the median reads and genes per spot, mapping confidence of transcripts to the genome and total number of detected genes, among other metrics.

Supplementary Table 3S: Tabular representation of pathways classified as overrepresented within the endothelial cluster data, as defined by the 'gprofiler' R package.

Supplementary Table S4: Tabular representation of pathways classified as overrepresented within the smooth muscle cluster data, as defined by the 'gprofiler' R package.

Supplementary Table S5: Tabular representation of pathways classified as overrepresented within the fibroblast cluster data, as defined by the 'gprofiler' R package.

Supplementary Table S6: Differentially expressed genes identified in the unassigned cluster subset by comparison of control tissue (static conditions) and flow tissue (tissue subjected to acute arterial haemodynamic perfusion).
