# Supplementary material for: Spatial Transcriptomic Profiling of Human Saphenous Vein Exposed to Ex Vivo Arterial Haemodynamics—Implications for Coronary Artery Bypass Graft Patency and Vein Graft Disease
Source: Int J Mol Sci. 2024 Sep 26;25(19):10368. doi: 10.3390/ijms251910368 (PMC11476946; doi:10.3390/ijms251910368)
Supplement: Supplementary file 1 [file ijms-25-10368-s001.zip › Supplementary Figures.pptx]

## Slide 1
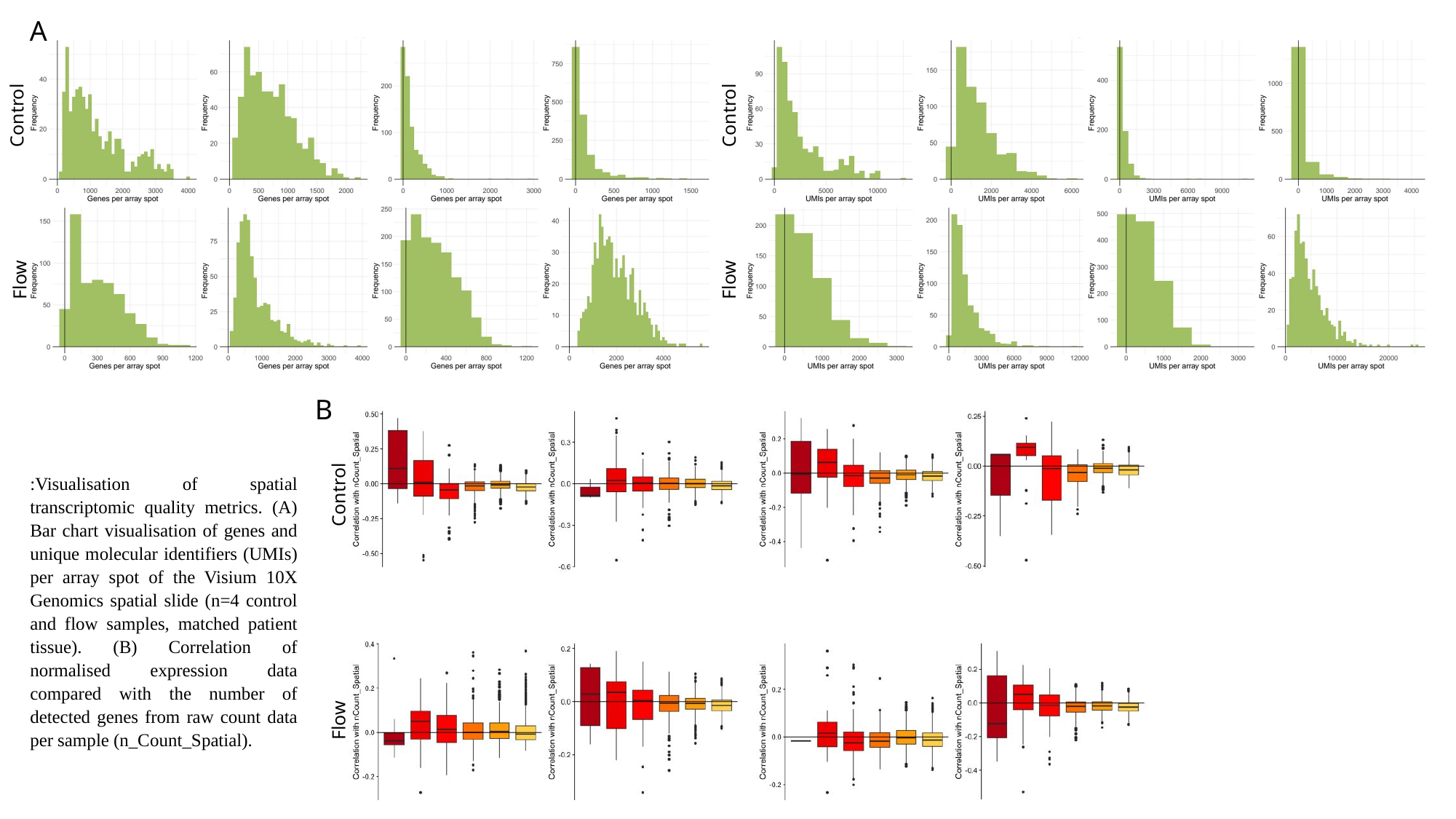

A
Control
Control
Flow
Flow
B
:Visualisation of spatial transcriptomic quality metrics. (A) Bar chart visualisation of genes and unique molecular identifiers (UMIs) per array spot of the Visium 10X Genomics spatial slide (n=4 control and flow samples, matched patient tissue). (B) Correlation of normalised expression data compared with the number of detected genes from raw count data per sample (n_Count_Spatial).
Control
Flow

## Slide 2
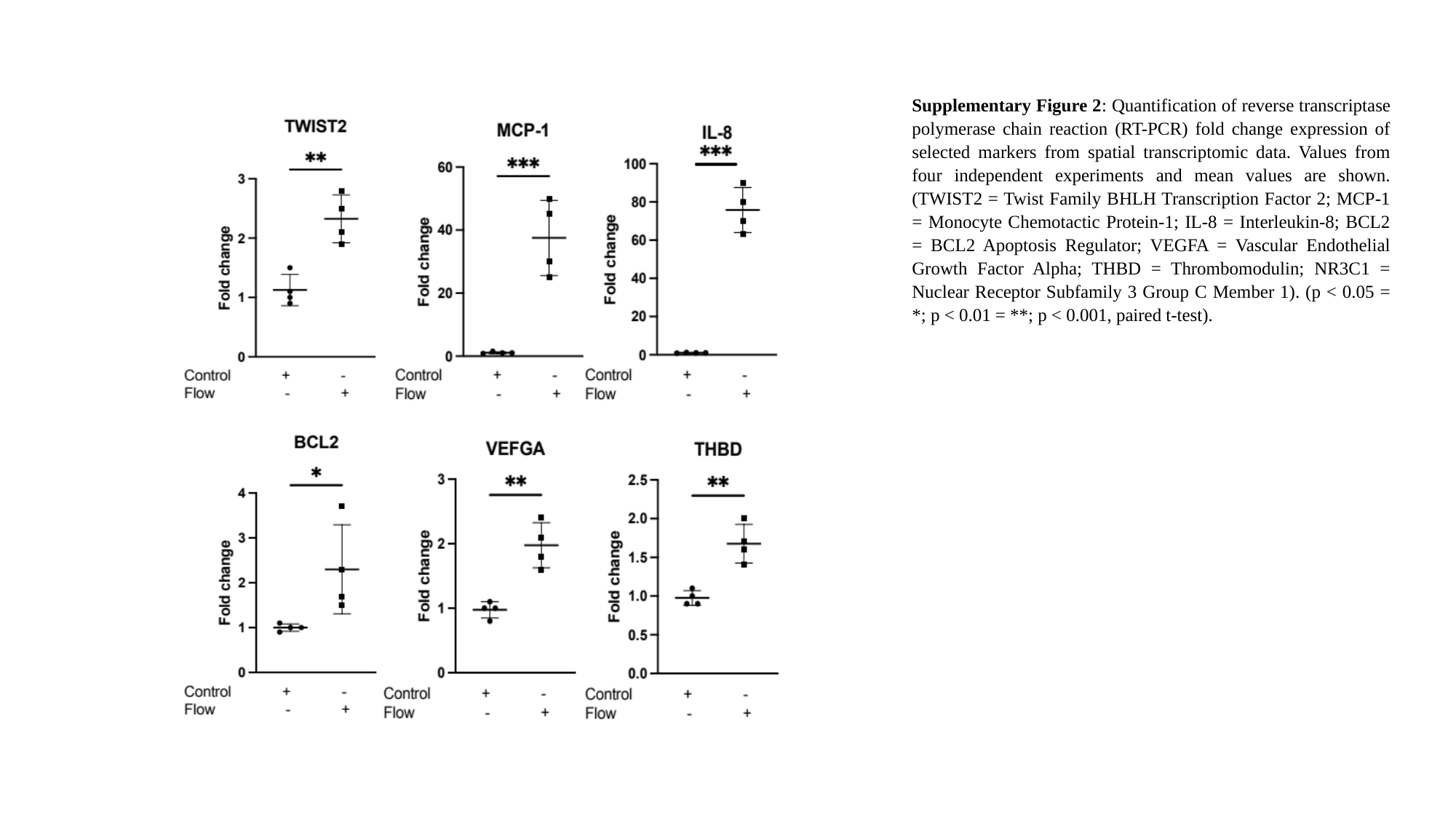

Supplementary Figure 2: Quantification of reverse transcriptase polymerase chain reaction (RT-PCR) fold change expression of selected markers from spatial transcriptomic data. Values from four independent experiments and mean values are shown. (TWIST2 = Twist Family BHLH Transcription Factor 2; MCP-1 = Monocyte Chemotactic Protein-1; IL-8 = Interleukin-8; BCL2 = BCL2 Apoptosis Regulator; VEGFA = Vascular Endothelial Growth Factor Alpha; THBD = Thrombomodulin; NR3C1 = Nuclear Receptor Subfamily 3 Group C Member 1). (p < 0.05 = *; p < 0.01 = **; p < 0.001, paired t-test).

## Slide 3
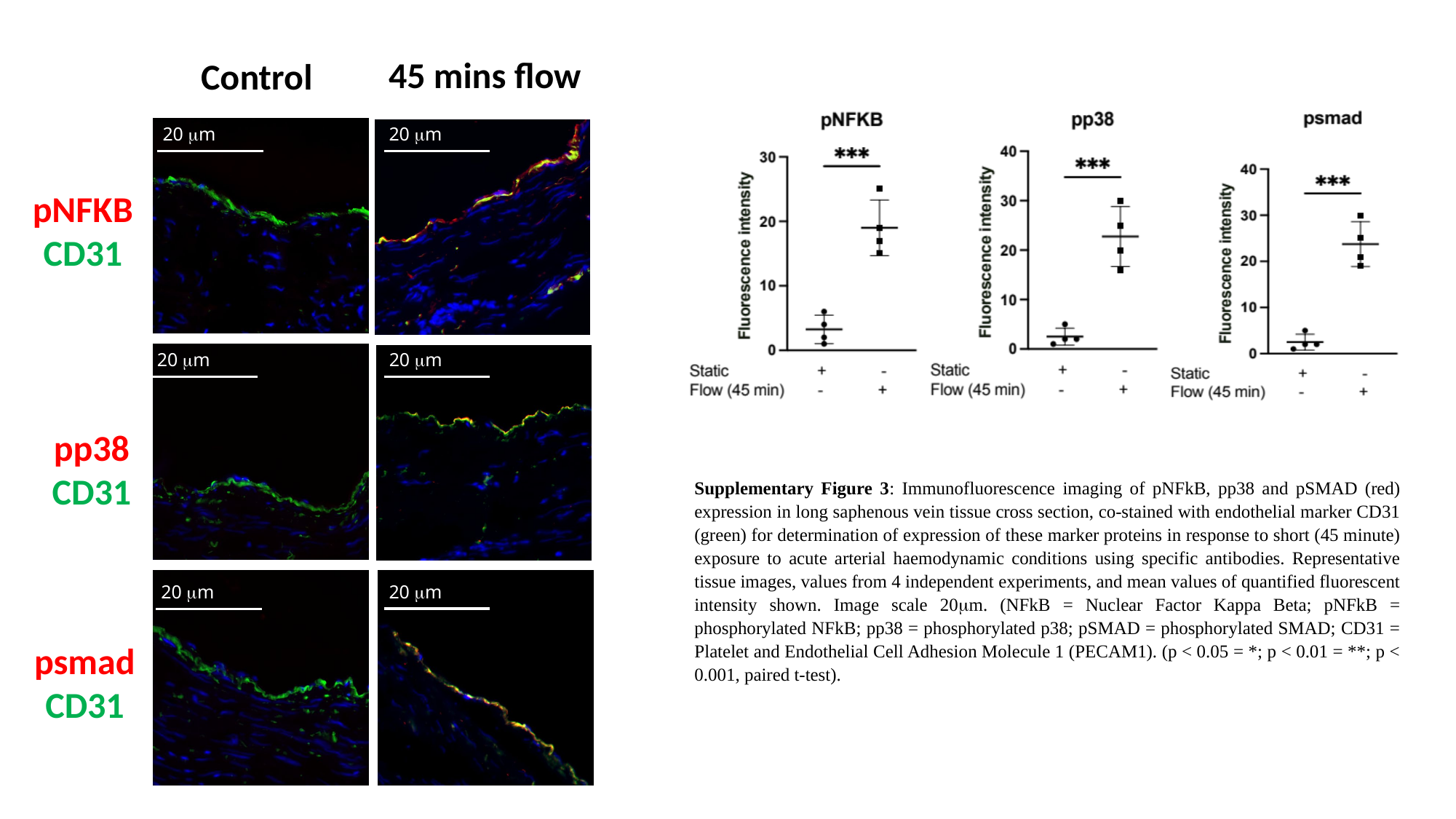

45 mins flow
Control
20 μm
20 μm
pNFKB
CD31
20 μm
20 μm
pp38
CD31
Supplementary Figure 3: Immunofluorescence imaging of pNFkB, pp38 and pSMAD (red) expression in long saphenous vein tissue cross section, co-stained with endothelial marker CD31 (green) for determination of expression of these marker proteins in response to short (45 minute) exposure to acute arterial haemodynamic conditions using specific antibodies. Representative tissue images, values from 4 independent experiments, and mean values of quantified fluorescent intensity shown. Image scale 20μm. (NFkB = Nuclear Factor Kappa Beta; pNFkB = phosphorylated NFkB; pp38 = phosphorylated p38; pSMAD = phosphorylated SMAD; CD31 = Platelet and Endothelial Cell Adhesion Molecule 1 (PECAM1). (p < 0.05 = *; p < 0.01 = **; p < 0.001, paired t-test).
20 μm
20 μm
psmad
CD31
